# When work–family guilt becomes a women's issue: Internalized gender stereotypes predict high guilt in working mothers but low guilt in working fathers

**DOI:** 10.1111/bjso.12575

**Published:** 2022-09-13

**Authors:** Lianne Aarntzen, Belle Derks, Elianne van Steenbergen, Tanja van der Lippe

**Affiliations:** ^1^ University of Twente Enschede The Netherlands; ^2^ Utrecht University Utrecht The Netherlands

**Keywords:** fathers, gender stereotypes, guilt, mothers, work–family conflict

## Abstract

Gender stereotypes prescribe mothers, but not fathers, to prioritize their family over their work. Therefore, internalization of gender stereotypes may predict higher guilt among mothers than fathers in situations in which they prioritize their work over their family. Study 1 (135 mothers and 116 fathers) indeed revealed that the stronger fathers' implicit gender stereotypes (measured with a gender‐career implicit association task) the less guilt fathers reported in a fictitious work‐interfering‐with‐family situation. Although mothers on average reported higher guilt than fathers, this effect was not moderated by their implicit gender stereotypes. Study 2 (daily diary study among 105 mothers), however, did reveal evidence for the moderating effect of implicit gender stereotypes on working mothers' guilt. The stronger mothers' implicit gender stereotypes the more work–family conflict and guilt they reported on days that they worked long hours. These results show that implicit gender stereotypes shape how parents feel about their work–family choices.

## BACKGROUND

Gender differences in work–family decisions often develop or are magnified when men and women become parents. For example, after having a baby, mothers often decrease their paid working hours and increase the time they spend on care tasks at home (Nomaguchi & Milkie, 2003; Paull, 2008). Fathers, on the other hand, hardly change their time allocation or even tend to increase their working hours (Choi et al., 2005; Kaufman & Uhlenberg, 2000).

Insight into reasons why these gendered work–family decisions persist is offered both by the literature on gender inequality in the work domain and by the literature on gender inequality in the home domain. For example, mothers who return to work after childbirth often receive unequal employment arrangements, such as being assigned less interesting or complex tasks (Yerkes et al., 2017), and career‐focussed mothers are more negatively evaluated by their colleagues than family‐focussed mothers (Morgenroth & Heilman, 2017). Furthermore, men are stigmatized as ‘unmanly’ when they shoulder a large proportion of the domestic responsibility (Chaney et al., 2019) and women as ‘cold’ and ‘selfish’ if they do not prioritize their care tasks (Haines & Stroessner, 2019), and women (but not men) are often held accountable for managing the household (Ciciolla & Luthar, 2019). Such prescriptive stereotypes on how fathers and mothers ‘should’ behave may push mothers into reducing their working hours and taking on the brunt of domestic work.

Although there are clear inequalities in the treatment, evaluations and outcomes of working mothers and fathers, parents themselves and the people around them often explain their gendered work–family decision as a ‘free choice’ (Stephens & Levine, 2011). For example, a survey demonstrates that many more women than men give up their careers, citing as the primary reason that they wish to increase the time that they have with their family (Hewlett & Luce, 2005). Furthermore, recent research shows that mothers experience more guilt than fathers when their work affects their family (Borelli et al., 2017), and that this guilt, in turn, predicts the gendered work–family decisions that parents make (Aarntzen et al., 2019). A crucial question, therefore, is why mothers are more prone to experience this work–family guilt than fathers. Is this because men and women want different things, or do societal norms push fathers and mothers into making different work–family choices?

We propose that parents' internalization of gender stereotypes linking mothers to caregiving and fathers to work (Morgenroth & Heilman, 2017; Park et al., 2010) can at least in part account for gender differences in guilt. The extent to which parents internalize these gender stereotypes and associate men more strongly with work and women with family might predict how guilty they feel in situations in which their work interferes with their family. Our main hypothesis is that the stronger parents themselves implicitly associate women with family and men with work, the more guilt mothers experience, and the less guilt fathers experience when their work interferes with their family time.

### Gender stereotypes

Although the gender landscape has changed over the past few decades (e.g. women today are well represented in educational institutes and the workforce; European Commission, 2021), mothers still perform most of the childcare and household tasks at home while fathers work more hours outside the home and earn most of the household income (European Commission, 2021; Intermediair, 2022; Pew Research Center, 2021). Although, today, it is considered acceptable that mothers earn some of the family income and fathers do some of the household and childcare when parents prioritize the gender a‐typical domain (family tasks for men, work tasks for women), backlash is likely to occur (Berdahl & Moon, 2013; Morgenroth & Heilman, 2017). Social role theory posits that the reason for this is that the different roles that men (being the main earner) and women (being the main caregiver) occupy, cause society to also develop different expectations of what men and women are like (i.e. women are expected to be warm and caring and men agentic and strong; Eagly et al., 2000).

While blatant gender stereotypes become less common (Dovidio et al., 2017), people all over the world still associate mothers more easily with family and fathers more easily with work than the other way around (Park et al., 2010; Project Implicit, 2022). Interestingly, even individuals who explicitly state that they strive towards gender equality are often found to more easily associate men with the role of breadwinner and women with the role of caregiver (Dovidio et al., 2001). The explanation for this paradox is that implicit stereotypes are not so much based on people's explicit attitudes, but on what they see in their direct environment and society more generally (Nosek et al., 2002).

Recently, several questions have been raised about the exact nature of implicit stereotypes. Are implicit stereotypes stable or susceptible to change? Are they mere associations or do they indicate an unconscious attitude towards different social groups (Payne et al., 2017)? Although the debate about what implicit gender stereotypes actually are continues, there is ample evidence that shows that implicit stereotypes predict how individuals perceive and judge others (Denessen et al., 2020; Park et al., 2010). Meta‐analytical research even shows that such implicit measures have higher predictive value on sensitive themes such as racial and gender stereotypes, (Denessen et al., 2020; Greenwald et al., 2009), because they prevent social desirable answers to a certain extent and can capture associations that participants are not fully aware of (Gawronski & De Houwer, 2014).

Implicit measurements of stereotypes are thus invaluable for predicting how individuals make moral judgements about others. To illustrate, one study showed that participants' implicit associations between fathers with work and mothers with family predicted their explicit judgements on how they think that parents should resolve a situation in which work and family demands are incompatible (i.e. work–family conflict; Park et al., 2010). When participants had stronger implicit associations that paired women with childcare and men with work, they also agreed more with statements indicating that the mother should resolve an imaginary work–family conflict situation by putting family first, while the father should resolve the identical conflict situation by putting work first. This effect existed over and above participants' explicit gender attitudes. Another study using hypothetical divorce cases showed that the degree to which participants associated women more with warmth‐related traits than men predicted how likely they were to give greater custody allocation to mothers than fathers (Costa et al., 2019).

In the current research, instead of focussing on how implicit gender stereotypes predict moral judgements about others, we examine how implicit gender stereotypes predict moral judgement about the self. Specifically, we examined how the extent to which parents implicitly associated men with work and women with family (implicit gender stereotypes) predicted their work–family guilt.

### How implicit gender stereotypes predict guilt

Guilt is a self‐evaluative, moral emotion that individuals experience when they condemn their own actions (Tangney et al., 2007). As a result of gender stereotypes that prescribe fathers to prioritize breadwinning and mothers to prioritize caregiving, mothers might be more prone to avoid situations in which they would need to prioritize their work over their family than men. Consequently, mothers may be more prone than fathers to feel guilty when their work interferes with their family (i.e. work‐to‐family conflict; WFC, Aarntzen, 2020). Previous research indeed found that in the same WFC situation, mothers report experiencing more guilt than fathers do (Aarntzen, 2020). However, the question remains if these gender differences in guilt result in part from parents' internalized gender stereotypes.

To explain gender differences in work–family guilt, previous research mainly focussed on explicit gender beliefs (Korabik, 2015; Livingston & Judge, 2008; Martínez et al., 2011), that is on how parents' explicit opinions about appropriate gender roles predict how much guilt they experience when their work interferes with their family. As shown in Korabik's overview (2015), this literature is rife with inconsistent results. For example, some researchers found that individuals with more explicit egalitarian attitudes feel more work–family guilt, irrespective of their gender (Livingston & Judge, 2008), while other researchers found that especially women with traditional attitudes feel guilty when their work interferes with their family (Korabik, 2015), and again others found that men with more traditional attitudes feel less guilty when their work interferes with their family than men with more egalitarian attitudes, while for women, the traditionality of their attitudes does not predict their work–family guilt (Martínez et al., 2011).

With the current study, we are the first to focus on implicit rather than explicit gender stereotypes and examine how these implicit stereotypes predict parents' experience of WFC and work–family guilt. We choose to do so because while nowadays explicit societal norms emphasize primarily the importance of equality between genders (Bolzendahl & Myers, 2004), more subtle, implicit gender stereotypes (e.g. schools often call the mother, not the father when the child is ill) still subtly convey that men should prioritize their breadwinning role and women their caregiving role. If internalization of such implicit stereotypes is partially predictive of parents' work–family guilt, this is crucial in understanding why mothers and fathers often make traditional work and family decisions and how to intervene. Previous research showed that higher work–family guilt in mothers is related to gendered behaviours: mothers who feel more guilty think more about reducing their working hours, reducing the time they planned for themselves, and plan to reserve more time and energy for their children in the future (Aarntzen et al., 2019). Therefore, it is important to explore how implicit gender stereotypes may predict higher feelings of guilt in mothers and lower feelings of guilt in fathers. This may generate insight into why mothers more often take on the majority of childcare tasks than their male counterparts and why women but not men often scale down their paid work, redefine their job or even leave the career field after becoming a parent (Belkin, 2003; Endendijk et al., 2018).

### The present research

The current studies aimed to examine whether implicit gender stereotypes predict parental guilt when parents prioritize their work over their family. In Study 1, we used a fictitious WFC situation to test whether the relationship between gender and work–family guilt was moderated by parents' implicit gender stereotypes. We expected that the stronger the implicit gender stereotypes of mothers (i.e. the more mothers implicitly associate women with the family domain and men with the work domain), the more work–family guilt they would report (H1). By contrast, for fathers, we expected that the stronger their implicit gender stereotypes, the less work–family guilt they would report (H2). Focussing on mothers, in Study 2, we used a daily diary design to test whether day‐to‐day fluctuations in mothers' working hours predicted their experience of WFC and work–family guilt and how this depended upon their implicit gender stereotypes. We expected that the stronger mothers' implicit gender stereotypes the more they would experience working long hours as a conflict and would feel guilty about this (H3). In both studies, implicit gender stereotypes were measured with the gender–career implicit association test (IAT; Nosek et al., 2002), which is considered a valid way to assess these associations (Lane et al., 2007).

## STUDY 1

In Study 1, we asked participants to consider an imaginary WFC situation, and indicate how guilty they expected they would feel in that situation. We chose to let participants *imagine* a situation in which they prioritized work over family to be able to pinpoint if gender differences in guilt occur when the work–family context is identical for mothers and fathers. This way, we could investigate whether fathers and mothers anticipate experiencing an objectively identical work–family situation differently depending on their implicit gender stereotypes.

### Participants and procedure

Participants were 251 parents (53.8% women), who were recruited in a Dutch Science Museum in a free‐play area and brought to a separate testing room. All parents met our participant criteria: they were in a heterosexual relationship, had a paid job and had at least one child. After providing informed consent, they completed a computerized questionnaire and then an implicit association test. Parents had on average 2.24 children (*SD* = 0.76), and the mean age of their (youngest) child was 8.00 (*SD* = 3.14).^1^ When asked about their highest educational level, 58.9% of the participants indicated university or higher vocational education, 28.3% indicated lower vocational education, 9.6% indicated high school and one participant (0.4%) indicated primary school. Fathers worked on average 42.32 h (*SD* = 8.18), and mothers worked on average 27.21 (*SD* = 9.41) a week. This is in line with the Dutch average of working hours among fathers (i.e. 35 hours a week) and mothers (i.e. 26 h per week), although fathers in our sample indicated to work somewhat more than the average Dutch father (Herter, 2022). A large part of our data consisted of couples (i.e. 68 dyads, which is 54.18% of our total sample).

### Measures

#### Implicit gender stereotypes

Participants' implicit gender stereotypes were assessed with a gender–career implicit association task (IAT; Nosek et al., 2002). This task measures the strength of each participant's associations between gender (male and female names) and family versus work‐related words. For more details on the measurement, see Supporting Information.

#### Work–family guilt

Participants were instructed to imagine a situation in which they could not stay home from work to take care of their sick child because they had to go to work, while their partner was able to stay home with the child. Then, we measured work–family guilt by asking participants how they would feel in this situation using three items (i.e. ‘I would feel fine’, ‘I would feel bad’, ‘I would feel guilty’; *α* = .86; the first item was reverse coded).

#### Work–family choice

To measure how participants would normally act in a situation in which their child was sick while they were very busy at work, they were asked to rate how they would solve this situation in real life. There were four options: (1) ‘I cancel work and stay at home’ (coded as prioritizing family; *n* = 104, 41.4% of participants) (2) ‘My partner cancels work and stays at home’ (coded as prioritizing work; *n* = 38, 15.1% of participants), (3) ‘We both go to work and our child stays with the babysitter or goes to the daycare’ [also coded as prioritizing work; *n* = 35, 13.9% of participants] or (4) ‘Other choice’, in which participants could fill out their own answer (*n* = 74, 29.5% of participants). Some of the ‘other choice’ responses could be recoded into prioritizing work (*n* = 31; e.g. ‘the grandparents babysit’) or prioritizing family (*n* = 4, e.g. ‘I cancel work when my child really has the flu’). The remaining ‘other choice’ responses fell into three categories: (1) ‘Working from home’ (*n* = 8), (2) ‘It depends’ (*n* = 27) and (3) ‘Children are old enough to stay at home alone’ (*n* = 1) Three responses were ‘empty’ and could therefore not be recoded. Data analyses focussed on responses that could be categorized (including recoded responses) as prioritizing work (*n* = 108, 49.1%) or prioritizing family (*n* = 108, 50.9%).

### Results

Tables 1 and 2 shows the sample characteristics and correlations between the measures (Table 1 for mothers and fathers separately, and Table 2 for the whole sample). The relatively high mean on guilt (i.e. 4.51 on a 7‐point scale) shows that, on average, parents anticipated feeling guilty in the described WFC situation. Furthermore, results on the IAT showed that, on average, both male and female participants associated work more with men and family more with women (i.e. a traditional view; average IAT *d*‐score = 0.41, *SD* = 0.39). The correlations (see Tables 1 and 2) revealed that the fewer hours parents worked in real life, the more guilt they anticipated feeling in the described imaginary WFC situation.

**TABLE 1:** Descriptive statistics and bivariate correlations among measures in Study 1 for men and women seperately

	Women	Men	Correlations			
	*M* (*SD*)	*M* (*SD*)	1	2	3	4
1. Working hours	27.21 (9.41)^a^	42.32 (8.18)^b^	–	−.03	.22*	−.09
2. Age youngest child^a^	8.14 (3.42)^a^	7.83 (2.79)^a^	.03	–	−.07	.20
3. IAT	0.44 (0.37)^a^	0.36 (0.40)^a^	.02	−.09	–	−.23*
4. Guilt	4.70 (1.76)^a^	4.29 (1.62)^b^	−.16±	.01	.04	–

*Note*: Correlations for women are presented below the diagonal and for men above the diagonal. Means in the same column with different subscripts differ (marginally) significant from each other (working hours *p* < .01; Guilt *p* = .06). IAT = implicit association test D‐score: positive scores represent a stronger association between ‘work and men’ and between ‘family and woman’.

^a^ These correlations are based upon a subset of participants (64%), since we do not have children's age for some participants. ±*p* < .10; *p < .05; **p < .01.

**TABLE 2:** Descriptive statistics and bivariate correlations among measures in Study 1

	*M* (*SD*)	Correlations				
		1	2	3	4	5
1. Gender	0.54 (0.50)	1				
2. Working hours	34.20 (11.60)	−.65**	1			
3. Age youngest child^a^	8.00 (3.14)	.05	−.03	1		
4. IAT	0.41 (0.39)	.10	.01	−.07	1	
5. Guilt	4.51 (1.71)	.12±	−.18**	.09	−.07	1

*Note*: IAT = implicit association test D‐score: positive scores represent a stronger association between ‘work and men’ and between ‘family and woman’.

^a^ These correlations are based upon a subset of participants (64%), since we do not have children's age for some participants. ±*p* < .10; *p < .05; **p < .01.

### Data analysis

Since a large part of our data consists of couples, our dataset possibly did not meet the GLM assumption of independent observations (Hox et al., 2017). Therefore, we first determined whether the data needed to be analysed with multilevel analysis. The intraclass correlation coefficient (ICC) was 0.20 for work–family guilt, suggesting that the couple level explained 20% of the variance in work–family guilt. In line with the recommendations of Bickel (2007) and Maas and Hox (2005) to take clustering into account if ICC is larger than .10 to prevent inflated type 1 error rates, we performed multilevel analysis using maximum likelihood estimation. Specifically, we estimated a multilevel (i.e. random‐intercept, fixed slope) model in which we entered (1) *couple* as a cluster variable, (2) the main effects of *gender* and *implicit gender stereotypes* (mean‐centred) and (3) the interaction effect between *gender* and *implicit gender stereotypes*. Multilevel modelling is highly recommended for nested data (i.e. participants nested within couples), also when some participants are not nested (i.e. participants that participated without their partner; Sterba, 2017). Specifically, the multilevel analysis allowed us to control for the fact that some participants were nested in couples, but it also allowed us to maximize power as all our participants are used, including the non‐nested participants (i.e. fathers and mothers that participated without their partner) in contrast to other analyses techniques such as cluster‐based summary statistics. Furthermore, note that analysing the dyads from our sample with the actor–partner interdependence model, resulted in the same patterns and significance levels (see Supporting Information).

### Do implicit gender stereotypes predict higher work–family guilt in mothers and lower work–family guilt in fathers?

**FIGURE 1:**
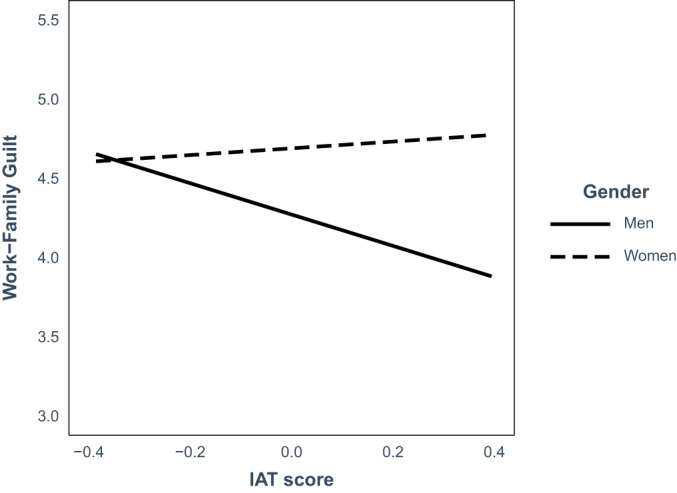
Parents' work–family guilt as a function of their gender and their gender–career implicit association test (IAT) score. IAT score was mean_centered. Lower scores on IAT indicate more egalitarian associations while higher scores indicate more traditional associations. *p < .05; **p < .01

Analyses revealed a main effect of gender (mothers experience more guilt than fathers; *B* = 0.42, *SE* = 0.20, *p* = .04, 95% CI [0.02; 0.81]) and a main effect of implicit stereotypes (the more parents have egalitarian gender stereotypes the more guilt they experience; *B* = −0.99, *SE* = 0.38, *p* = .01, 95% CI [−1.74; −.024]). Furthermore, analyses revealed a significant interaction effect between gender and implicit gender stereotypes on anticipated guilt (*B* = 1.20, *SE* = 0.55, *p* = .03, 95% CI [0.12; 2.28]; see Figure 1). To interpret this significant interaction, we tested simple slopes of parents with more egalitarian implicit gender stereotypes (−1 *SD*) and more traditional implicit gender stereotypes (+1 *SD*). Note that in our sample, these more egalitarian implicit gender stereotypes from minus 1 *SD* represent an IAT‐score of 0.02 which is classified as egalitarian and the more traditional implicit gender stereotypes represent an IAT score of 0.79 which is classified as a strongly traditional (Project implicit, 2022). When parents had more egalitarian implicit gender stereotypes, mothers and fathers did not differ in their work–family guilt (*t* = −.016, *p =* .88). However, when parents had more traditional implicit stereotypes mothers reported stronger guilt than fathers: *B* = 0.88, *SE* = 0.29, *p* = .003, 95% CI [0.31; 1.46]. Looking within gender, we found that men with more traditional stereotypes anticipated feeling less guilty in response to the scenario than men with more egalitarian stereotypes did (*B* = −0.99, *SE* = 0.39, *p* = .01, 95% CI [−0.23; −2.56]), whereas implicit gender stereotypes failed to predict anticipated guilt in mothers (*t* = 0.54*, p* = .59). This suggests that when parents are confronted with WFC, fathers with more traditional gender stereotypes are protected from feeling high work–family guilt than fathers with more egalitarian gender stereotypes and/or mothers (irrespective of the direction of mothers' implicit gender stereotypes).

### How does participants' own decision of prioritizing work or family in the proposed situation alter the results?

The effect of gender and gender stereotypes on work–family guilt may be different for parents whose preferred work–family choice is incongruent with this situation (i.e. parents who indicated that they would prioritize family while the scenario asks them to imagine that they would prioritize work) compared with parents whose work–family choice is congruent with this situation (i.e. prioritize work). For example, the latter could have imagined the situation more vividly as it represents a situation that happens in their real life. Therefore, we explored a three‐way interaction between gender, gender stereotypes and work–family choice (i.e. prioritize work or prioritize family) on work–family guilt. However, this three‐way interaction effect was not significant (*t* = 0.22, *p =* .83); therefore, we have no reason to assume that the relationship between gender and guilt is different for parents who indicated that in real life, they would prioritize family versus parents who indicated that in real life, they would prioritize work. For more explorative analyses on the effect of participants' preferred work–family choice, see Supporting Information.

### Conclusion study 1

Study 1 suggests that when parents are confronted with a situation in which they imagine prioritizing work over family, traditional implicit gender stereotypes that tie mothers to family and fathers to work, protect fathers from feeling guilty. Specifically, fathers with more traditional gender stereotypes (i.e. fathers who strongly associate women with childcare and men with work) anticipated feeling less guilty in the WFC situation than fathers with more egalitarian gender stereotypes did while these more egalitarian fathers did not differ in their anticipated guilt from mothers.

In contrast to our hypothesis, we did not find that mothers with more traditional implicit gender stereotypes anticipated more guilt than mothers with more egalitarian implicit gender stereotypes. One reason why implicit gender stereotypes may not have predicted guilt in mothers in this fictitious clear‐cut situation is that the experienced WFC was quite high for both egalitarian and traditional mothers (i.e. almost 60% of women indicated that in real life, they rather than their partner would stay at home when their child was sick). Hence, this situation may be unfit to differentiate between more traditional and more egalitarian women.

However, in real life, women may often encounter work–family situations that only some mothers interpret as WFC, and feel guilty about, while other mothers would not. For example, situations such as working overtime or not having the energy to cook a healthy meal when coming home from work may constitute more common situations for working mothers. In such situations, mothers with more traditional gender stereotypes may appraise this more as a work–family conflict and experience more guilt than mothers with more egalitarian gender stereotypes who may not even interpret such a situation as a conflict. Therefore, in Study 2, we examined how daily working hours predict the experience of WFC and guilt in working women. We expected that the same objective situation (i.e. work more than the usual 8‐h working day) is more quickly interpreted by traditional women as WFC than by egalitarian women, and consequently, traditional women may be more likely to experience guilt than egalitarian women. As such, in Study 2, we further explored how gender stereotypes may predict mothers' real‐life work–family experiences.

## STUDY 2

In Study 2, we performed a daily diary study to examine whether women's WFC experiences and feelings of guilt in real life are predicted by their implicit gender stereotypes.^2^ A first advantage of a daily diary design is that study variables are more reliably measured because retrospective bias is reduced and the study variables are measured multiple times. A second advantage is that the diary study allows us to investigate how work hours, WFC and guilt fluctuate across days for the average woman depending on her implicit gender stereotypes (i.e. test relationships within individuals; Ohly et al., 2010). Women who experienced a lot of WFC and guilt in the past may have adapted their working hours; therefore, we may not find the hypothesized relationships with a cross‐sectional study. However, with the daily diary design, we can still reliably test these relationships because we can examine how day‐to‐day fluctuations in working hours predict levels of WFC and guilt. Moreover, we can examine whether the strength of these relationships depends upon women's implicit gender stereotypes.

### Participants and procedure

We recruited 123 working mothers, who all had at least one child aged 13 years or younger via the personal network of nine undergraduate students who helped with data collection as part of their curriculum. Of these 123 participants, 105 participants were included in our analyses (these participants filled out the start questionnaire and at least one daily measure). Upon signing up for this study, mothers provided an email address to which we sent the online questionnaires and a cellphone number to which daily reminders were sent. On the first day, mothers provided informed consent and completed the implicit association task (IAT) and a 5‐minute online questionnaire in which background characteristics were assessed (e.g. age and working hours). Then, over eight consecutive days, mothers filled out a 5‐minute online survey in which we assessed their work hours, WFC and work–family guilt on that day. All daily questionnaires that were filled out on the day itself or before 10.30 the next morning were included in our analyses. All other diary entries were marked as missing. This resulted in 724 entries distributed with a mean of 6.90 entries per person (*SD* = 0.70).

Most participants were either married or cohabiting (98.09%) and had between 1 and 4 children (*M* = 2.01; *SD* = 0.74). The age of their youngest child (or only child) ranged between one and 13 years (*M* = 6.58; *SD* = 3.85). Mothers worked, on average, 28.66 hours per week (*SD* = 8.86). This is in line with the Dutch average of working hours among mothers (i.e. 26 hours per week; Herter, 2022; Van den Brakel & Merens, 2016) and almost the same as in Study 1 (i.e. *M* = 27.21). When asked about their highest educational degree, 74.4% of participants indicated university or higher vocational education, 22.9% indicated lower vocational education and 2.9% indicated high school.

### Measures

Implicit gender stereotypes were assessed on day 1. All other measures were assessed daily (8 consecutive days) with a single item using a 5‐point scale. We chose to use single‐item measures to optimize response rates (see Van Steenbergen et al., 2018 for a similar approach in a diary study).

*Implicit gender stereotypes (gender career‐IAT)*. Participants completed the same gender–career IAT test as in Study 1. Only in this study, participants completed the IAT online instead of in a testing room.

*Daily work–family conflict* was measured with the question ‘Did your work interfere with your activities at home today?’ (1 = Not at all ‐ 5 = Very much). Participants who did not go to work that day were still asked to estimate whether their work had an influence on their home activities.

*Daily work–family guilt* was measured with the item ‘If you think about how you combined work and family today, to what extent do you feel guilty towards your family? Today I feel…’ (1 = Not at all guilty ‐ 5 = Very guilty).

### Results

Table 3 presents all between‐participants correlations, means and standard deviations for the study variables (based on 8‐day aggregates for the daily measures). Similar to Study 1, participants associated work more with men and family more with women (i.e. a traditional view; average IAT *d*‐score = 0.51, *SD* = 0.27). This IAT score was not directly related to how much WFC and guilt mothers experienced over the week. However, IAT score was related to the daily work hours of mothers (i.e. more traditional mothers worked fewer hours). Furthermore, on average, participants reported low WFC during the week (mean of 2.15 on a 7‐point scale).

**TABLE 3:** Descriptive statistics and bivariate correlations among measures and background variables in Study 2

Measures	*M* (*SD*)	Correlations						
		1	2	3	4	5	6	7
1. Implicit association test^a^	0.50 (0.28)	–						
2. Workhours (general)	28.66 (8.86)	−.18	–					
3. Workhours partner (general)	38.84 (9.87)	.05	−.18	–				
4. Daily workhours	3.93 (2.31)	−.21*	.64**	−.16	–			
5. Daily work–family conflict	2.19 (0.76)	−.02	.29**	−.20*	.31**	–		
6. Daily work‐family guilt	1.69 (0.59)	.02	.33**	−.29**	.28**	.75**	–	
7. Age youngest child	6.58 (3.85)	.09	−.05	−.004	−.10	.02	−.05	–

*Note*: Daily workhours, daily work–family conflict and daily guilt are aggregates of variables that are measured daily.

^a^ IAT D‐score: Higher positive scores represent a stronger association between ‘work and men’ and between ‘family and woman’. *p < .05; **p < .01.

### Overview of analyses

**FIGURE 2:**
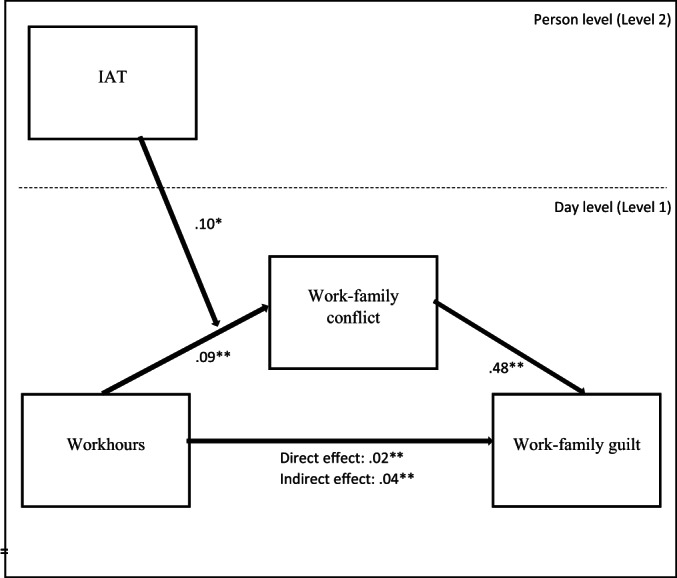
Multilevel moderated mediation model showing how daily work–family conflict mediates the relationship between daily workhours and daily work–family conflict and how implicit gender stereotypes (measured with the gender‐career implicit association test [IAT] on the person level) moderate the relationship between workhours and work–family conflict. *p < .05; **p < .01

We again had multilevel data (i.e. days nested within participants). Intraclass correlation coefficients (ICCs) were 0.19 for WFC and 0.22 for work–family guilt suggesting that a significant proportion of both variables could be explained by differences between participants (19% and 22% of the variance). Therefore, we again performed multilevel analysis using maximum likelihood estimation. We tested our entire model at once using the MLMED Matrix in SPSS (see Figure 2 for an overview of the model and the regression weights; Hayes & Rockwood, 2020).

### Do implicit gender stereotypes predict guilt among mothers?

**FIGURE 3:**
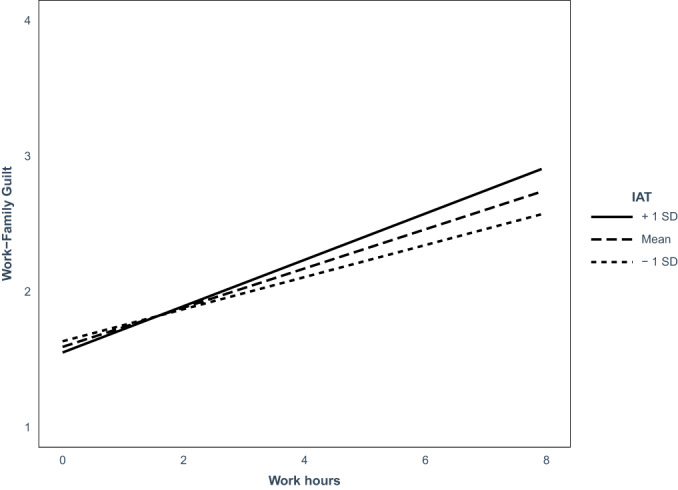
Interaction effect of implicit gender stereotypes (measured with the gender–career implicit association test [IAT]) and daily workhours on daily work–family conflict. The graph shows work–family conflict as a function of workhours per week and participants' IAT score. IAT score was mean_centered. Lower scores on IAT indicate more egalitarian associations while higher scores indicate more traditional associations. *p < .05; ns, not significant

Results of the analyses are presented in Table 4 and visualized in Figure 2. As expected, on days that mothers worked longer hours, they reported feeling more guilty and this relationship was mediated by mothers' experiencing more WFC on days that they worked longer hours. Furthermore, we found a significant interaction between working hours and implicit gender stereotypes on WFC (see Figure 3).^3^

**TABLE 4:** Relationships between daily working hours and guilt through WFC moderated by gender‐career implicit associations test score in study 2

Path/effect	*B*	*SE*	95% confidence interval
*c* (work hours → guilt)	0.02**	0.01	0.007, 0.039
*a* (work hours → WFC)	0.09**	0.02	0.039, 0.136
*b* (WFC → guilt)	0.48**	0.03	0.426, 0.534
*a* × *b* (mediation effect)	0.04**	0.01	0.019, 0.066
*m1* (work hours × IAT → WFC)	0.10*	0.04	0.017, 0.188

*Note*: WFC = work–family conflict, Guilt = work‐family guilt, IAT = implicit association test (higher positive scores represent a stronger association between ‘work and men’ and between ‘family and woman’). Estimates are unstandardized. *p < .05; **p < .01.

To interpret this significant interaction, we tested simple slopes of mothers with more egalitarian implicit gender stereotypes (−1 *SD*) and traditional implicit gender stereotypes (+1 *SD*). As expected, on days that mothers worked a full day (8 h) traditional mothers experienced more WFC than egalitarian mothers (*B* = 0.63, *SE* = 0.32, *p* = .046, 95% CI [0.02; 1.22]), but on a day off (0 h), traditional mothers and egalitarian mothers did not differ in their experience of WFC (*t* = −0.57, *p* = .57).

### Conclusion study 2

The results from Study 2 suggest that mothers interpret an objectively similar work day (working a full day) differentially depending upon how much they implicitly associate women with childcare and men with work. When working a full‐time day or more hours (≥8 h), mothers with more traditional associations interpret such a situation more as WFC and in turn experience more guilt than mothers with more egalitarian associations. Thus, although we could not differentiate between mothers with more traditional and more egalitarian implicit gender stereotypes in an imaginary WFC situation (Study 1) when looking at real‐life day‐to‐day fluctuations, mothers' implicit gender stereotypes predict their experienced WFC and guilt.

## GENERAL DISCUSSION

Substantial literature shows that the way that fathers and mothers combine their work and family remains heavily intertwined with their gender (European Commission, 2021; Stertz et al., 2017). This gender inequality may even be exacerbated by the unequal effect of the COVID‐19 pandemic on mothers and fathers (Myers et al., 2020; Yerkes et al., 2020). Higher levels of guilt among mothers than fathers (Borelli et al., 2017) result in mothers prioritizing their family caregiving tasks over their careers (Aarntzen et al., 2019). But why do mothers experience more work–family guilt than fathers? Different from popular media claiming that mothers have a ‘guilt gene’ or researchers hypothesizing that mothers feel more guilt than fathers because they themselves opine that a mother's primary role should be caregiving (e.g. see Korabik, 2015 for an overview of research on explicit gender beliefs and work‐family guilt), we show that internalized gender stereotypes pose an explanation for why work–family guilt is gendered. The more parents have internalized gender stereotypes (i.e., stronger associating men with work and women with family), the more work–family guilt mothers experience and the less work–family guilt fathers experience. Because implicit associations arise at least in part from what individuals see in their environment (Greenwald & Banaji, 2017; Payne et al., 2017), our results imply that gender differences in work–family guilt are at least in part a result of an environment in which women often are the primary caregiver and men often are the primary breadwinner.

The knowledge that internalized gender stereotypes can potentially cause high work–family guilt is important because work–family guilt is detrimental to the well‐being of working parents and contributes to gendered choices (Aarntzen et al., 2019). Taking into account how (1) work–family guilt may arise from gender stereotypes in our society and (2) gendered work–family choices may be a direct result of work‐family guilt, a vicious cycle may be at play: As parents often see in their direct environment or through media portrayals that women take on the role of primary caregiver and men of primary breadwinner, gender differences in work–family guilt arise, which results in gendered choices, and again leads to an environment that is typified by caregiving mothers and breadwinning fathers. Combined, these studies challenge the idea that mothers are generally more likely to experience work–family guilt than fathers and propose opportunities to break the vicious cycle in which gender stereotypes result in more guilt among mothers than fathers.

Although previous research indicated that men and women in real life often experience similar levels of WFC (Byron, 2005; Herman & Gyllstrom, 1977), we demonstrate that WFC in itself is a gendered experience. When men and women find themselves in the same work–family situation, their levels of WFC and guilt do differ depending upon their implicit gender stereotypes. This paper reveals that mothers with more traditional internalized stereotypes feel more guilty and are quicker to interpret a situation as work–family conflict compared with fathers or more egalitarian mothers. In Study 1, in which working fathers and mothers were asked to imagine the same situation in which they prioritized their work over their family, mothers anticipated feeling more guilty than fathers did. Interestingly, in such a WFC situation, fathers with more traditional gender stereotypes anticipated feeling less guilty than fathers with less traditional gender stereotypes. In a similar vein, in Study 2, we found that an objectively same work–family situation—working an 8‐hour day—was interpreted by mothers with more traditional gender stereotypes more as WFC, resulting in more guilt, than mothers with less traditional gender stereotypes. Our results suggest that in couples in which both parents have relatively egalitarian implicit gender stereotypes mothers and fathers may experience similar levels of guilt when their work interferes with their family. This in turn may lead parents to share work and family tasks more equally. Sharing care equally would then only be possible when feeling guilty is also shared.

If societal norms that place responsibility for family tasks on women are so strong, why then have large meta‐analyses not found consistent evidence for gender differences in levels of WFC or even found men to experience more WFC (Byron, 2005)? We propose that this is because in real life, the work–family division of men and women often differ a lot and are very likely attuned to the work–family guilt that mothers and fathers expect to experience. For example, on average, women spend less time on paid work and more on childcare than men do (European Commission, 2021). Possibly, the lack of difference in the levels of mothers' and fathers' WFC is due to the fact that the experience of WFC and the resulting guilt motivates parents to adapt their life in a way that they can feel more satisfied with how they combine work and family. For example, a working mother who often misses her children's bedtime ritual because of a long commute and feels high guilt about this may choose to take on a less challenging, less‐paid job because it is in her place of residence. As a result, mothers and fathers, on average, may experience similar amounts of work–family guilt, but when you compare their actual situations it becomes visible that to maintain a bearable level of WFC and guilt mothers work much fewer hours and spend more time with their children than fathers do. Therefore, research that investigates gender differences in WFC in ‘real life’ needs to take into account these differences in how fathers and mothers combine work and family to draw meaningful conclusions about differences in work–family conflict and work–family guilt between men and women.

### Limitations and future research

With these two studies, we are the first to provide evidence that objectively identical work–family circumstances can result in different levels of guilt in fathers and mothers depending on their internalized gender stereotypes. However, this research also has its limitations.

An obvious limitation is the correlational nature of both studies which makes that we cannot rule out the possibility of reversed causality. A question that may be raised is how the relationship between parents' working hours, their implicit gender stereotypes and their work–family guilt develops over time. Possibly, stronger traditional implicit gender stereotypes lead mothers to work fewer hours because they experience higher WFC and guilt if they would work more. Or in line with research showing that implicit associations can change through personal experiences (Endendijk et al., 2018), mothers who work fewer hours may develop more traditional associations resulting in enhanced work–family guilt in situations in which they still prioritize work. Note regarding this that in Study 2, we indeed found that stronger implicit gender stereotypes were related to mothers working fewer hours; however, when testing our hypothesis, we focussed on day‐to‐day fluctuations of women's working hours, and therefore, it was not problematic in this study to treat them both as independent variables. Still, it is likely that the relationship between implicit gender stereotypes, guilt, and choices is a recursive one where experiencing guilt about combining work and family among mothers (or the absence of guilt in fathers) may lead to more traditional work–family choices, which again may feed back into the development of stronger implicit gender stereotypes.

Furthermore, the current results indicate that high work–family guilt in mothers is a consequence of socio‐contextual circumstances rather than pre‐existing gender differences. Therefore, national culture might moderate the relationship between gender and guilt. The current studies were conducted in the Netherlands, a country that ranks high on explicit forms of gender equality (e.g. high participation of women in the workforce; World Economic Forum, 2020), but where at the same time, the traditional family model is still embraced (e.g. short paternity leave and well‐regulated part‐time work) and where people score relatively traditional on the Gender‐Career IAT compared with many other European countries (Project implicit, 2022). As such, gender differences in work–family guilt may be lower in parents who live in countries that have less gendered family norms, such as Iceland (a country that actively supports dual‐earner families with long paternity leave and high‐quality, heavily subsidized childcare) but may be higher in parents who live in countries that rank more gender unequal than the Netherlands on both more explicit (i.e. GEM index) and more implicit forms (i.e. IAT) of gender equality such as Hungary. Although a recent study indicated that while organizational cultures are predictive of fathers' and mothers' work–family guilt, national cultures are not (Aarntzen et al., 2021) therefore although, in need of further investigation, these studies may be generalizable to other countries.

Finally, one might wonder whether there are also contexts in which fathers experience more guilt than mothers. We propose that when parents fail to meet a parenting task that is typically associated with the father role (e.g. not being able to coach your child's soccer team or not putting up the baby stairgates on time) fathers indeed may experience more guilt than mothers, especially fathers with strong implicit gender stereotypes. Future research is needed to further explore WFC situations in which guilt may be especially high in fathers with more traditional implicit gender stereotypes (compared with mothers or fathers with more egalitarian implicit gender stereotypes).

### Implications

The current research has several theoretical implications. First, results imply that implicit associations can be an important predictor of emotions. Specifically, we found that associations between career, family and gender predict how parents interpret an objective same situation (going to work when your child is ill, working more than an 8‐hour day). This aligns with dual‐system models (e.g. reflective‐impulsive model; Strack & Deutsch, 2004) that suggests that to predict human ‘behaviour’, it is crucial to examine processes that operate beyond awareness and personal control. Furthermore, this supports stereotype internalization theories (Bonnot & Croizet, 2007) showing that the more parents have internalized women–caregiver, and men–breadwinner associations, the more they experience emotions along gender‐stereotypical lines. Finally, the current research demonstrates that work–family conflict is a subjective experience. This builds upon the transactional model of stress (Lazarus & Folkman, 1984), a theory that states that events are not stressful in themselves but become less or more stressful by how individuals appraise the situation. In line with this theory, we show that parents experience an objective same work–family situation differently depending upon their internalized gender norms, exposing an important mechanism that explains gender differences in fathers' and mothers' work–family experiences.

From our studies, also practical implications follow. We show that one way to promote equal work–family experiences for parents is to decrease traditional’ implicit associations as this may take away the gendered aspect of feeling guilty. Although implicit associations may not be easily malleable, a recent longitudinal study among parents of young children did suggest that parents' implicit gender associations may change as a function of their personal experiences (Endendijk et al., 2018). Although in need of further research, this provides promising possibilities for interventions. For example, making parents aware of their implicit associations and exposing them frequently to caregiving fathers and working mothers (e.g. reading counter stereotypical books) may decrease high guilt in mothers with more traditional associations and increase guilt in fathers with more traditional associations. Similarly, by encouraging parents to enact more egalitarian behaviours themselves (e.g. by having fathers more often take the responsibility when a child is sick), implicit gender stereotypes of both parents may become more egalitarian over time.

## CONCLUSION

Gender stereotypes in our society of ‘men as breadwinners’ and ‘women as caregivers’ shape how we evaluate fathers' and mothers' choices and behaviours (i.e. a mother who prioritizes work is seen as a bad parent; Okimoto & Heilman, 2012). Our research highlights that these gender stereotypes do not only shape evaluations of others but also shape how parents themselves feel about their work–family choices. Stronger internalization of gender stereotypes, tying mothers to family and fathers to work, predicted high work–family guilt among working mothers and low work–family guilt among working fathers. Guilt may underlie gendered work–family decisions, making mothers want to work less and invest more time in their parenting (Aarntzen et al., 2019). At the same time, low guilt in fathers may allow them to keep prioritizing work. Hence through work–family guilt, internalized gender stereotypes make it difficult for mothers to focus on their careers next to their family while they make it easier for fathers to solely focus on their careers. To reach gender equality in work and family roles, taking away the gendered aspect of feeling guilty when parents' work interferes with their parenting tasks is an important first step.

## AUTHOR CONTRIBUTIONS

**Lianne Aarntzen:** Conceptualization; data curation; formal analysis; investigation; methodology; project administration; resources; software; validation; visualization; writing – original draft; writing – review and editing. **Belle Derks:** Conceptualization; funding acquisition; methodology; supervision; writing – review and editing. **Elianne van Steenbergen:** Conceptualization; methodology; supervision; writing – review and editing. **Tanja van der Lippe:** Conceptualization; methodology; supervision; writing – review and editing.

## CONFLICT OF INTEREST

All authors declare no conflicts of interest.

## FUNDING INFORMATION

This work was supported by an NWO VIDI grant (016.155.391) awarded to B. Derks.

## Supporting information


Appendix S1

## Data Availability

The data of Study 1 and Study 2 are not made publicly available yet because data of both studies are still used for another research. After this research is completed, the data sets will be publicly archived in a secure public data repository such as DataverseNL.

## Footnotes

^1^
Due to a programming error in display logic, we miss the age of the (youngest) child when parents have two or more children of the same gender. Therefore, mean and *SD*'s of age (youngest) child are based on only 168 participants (66.4%).

^2^
This study was part of a larger research project. Another part of this dataset is reported in Aarntzen et al., 2019 and another is still being analyzed. In this paper, we only reported the measures relevant to answering the current research question. More details about other measurements and research questions in the larger research project are available from the first author.

^3^
Since there was a very high correlation between WFC and guilt, these concepts may not be statistically different. Therefore, we also tested the interaction between working hours and gender stereotypes on the aggregated variable of WFC and guilt (i.e., computing a mean‐score of the WFC item and the guilt item). With this aggregated concept, we also found a significant interaction effect between gender stereotypes and working hours that could be interpreted along the same lines as the interaction between gender stereotypes and working hours on WFC. Because we think that WFC and guilt are theoretically different concepts (e.g., when measuring the well‐validated concept of WFC, guilt is never included as an item) and because we also measured work‐family guilt independently of WFC in Study 1, we decided to keep the concepts separate in Study 2.
