# Supplementary material for: When work–family guilt becomes a women's issue: Internalized gender stereotypes predict high guilt in working mothers but low guilt in working fathers
Source: Br J Soc Psychol. 2022 Sep 13;62(1):12–29. doi: 10.1111/bjso.12575 (PMC10087844; doi:10.1111/bjso.12575)
Supplement: Supplementary file 1 — Appendix S1 [file BJSO-62-12-s001.zip › BJSO_12575_Online supplement.docx]

**Online supplement**

**Study 1: Method**

***Gender Career IAT***

In our Gender-Career IAT participants are asked to categorize Dutch male names (i.e., ‘Thomas’, ‘Daan’, ‘Tim’, ‘Lucas’, ‘Stijn’, ‘Luuk’, ‘Sven’, ‘Niels’, ‘Jasper’), Dutch female names (i.e., ‘Anna’, ‘Sanne’, ‘Julia’, ‘Emma’, ‘Sophie’, ‘Lisa’, ‘Lotte’, ‘Eva’, ‘Lieke’), family words (i.e., Dutch translations of the following words: ‘children’, ‘parents’, ‘family-members’, ‘diapers’, ‘caring’, ‘cooking’, ‘comforting’, ‘baby’) and career words (i.e., Dutch translations of the following words: ‘management’, ‘professional’, ‘business’, ‘salary’, ‘office’, ‘career’, ‘promotion’, ‘job’) within the right category.

The task consisted of congruent blocks in which participants categorized female names and family items by pressing one key and male names and work items by pressing another key and incongruent blocks in which participants categorized male names and family items by pressing one key and female names and work items by pressing another key. To reduce possible order effects, the presentation of congruent and incongruent blocks, as well as the presentation of the names and items, were in a random order (all names, items, and blocks were presented once). We used the improved scoring algorithm of Greenwald et al. (2003) to calculate one IAT score per participant. Higher positive scores represent more traditional views (i.e., stronger associations between men and career and between women and family), 0 represents an egalitarian view (i.e., associate women and men equally strong with family and career), and higher negative scores represent stronger counter-stereotypical gender views (i.e., stronger associations between women and career and between men and family).

**Study 1: Results**

***Is guilt predicted by the implicit gender stereotypes of one’s partner?***

Research shows that parents’ work-family decisions are interdependent. For example, after childbirth, women who have partners with more egalitarian attitudes, take shorter maternal leaves and decrease their working hours to a lesser extent compared to mothers whose partner have more traditional attitudes (Sterz, Grether, & Wiese, 2017). Taking advantage of our dyadic data, we also explored whether partners’ implicit gender stereotypes predict parental guilt over and above the effect of one’s own implicit gender stereotypes by using the actor-partner interdependence model (APIM). Note that we only use a subset of the data in which both partners participated in the research (N_participants_ =136, N_dyads_ =68, 54.18%). The results of these analyses are summarized in Figure 1 (from supplement). Adding to the robustness of our findings, we again showed that fathers’ implicit gender stereotypes predicted how guilty they felt when they would not stay at home with their sick child and that mothers’ implicit gender stereotypes did *not* predict how guilty mothers feel when they would not stay at home with their sick child. With respect to the interdependence between couples’ gender stereotypes and each partner’s work-family guilt, analyses showed that partners’ IAT scores were positively correlated but that fathers’ implicit gender stereotypes did not predict mothers’ guilt and mothers’ implicit gender stereotypes did not predict fathers’ guilt.

***How does participants’ own decision of prioritizing work or family in the proposed situation alter the results?***

Participants were asked how they normally would solve a situation in which their child is sick while they are busy at work. Including only the recoded responses (i.e., recoded responses fell in two categories: prioritizing work or prioritizing family), 49.1% of participants reported that they indeed would most likely go to work while their partner or a babysitter stays with the sick child, while 50.9% reported that they would most likely stay at home themselves. A multilevel logistic regression analysis revealed that the more guilt participants anticipated to feel in this situation the higher the odds (1.30 times more likely) that participants reported to stay at home in this situation, Wald χ2(1, 9.42) = 1.30, 95% CI [1.10, 1.54], *B* = 0.26, *S.E*. = 0.08, *z* = 3.07, *p* = .002). An ANOVA confirmed that parents who would prioritize work (i.e., whose work-family choice was congruent with the proposed situation) reported lower guilt (*M* = 4.23, *SD* = 1.74) than parents who would prioritize family (i.e., whose work-family choice was incongruent with the proposed situation; *M* = 4.96, *SD* = 1.60, *95% CI* [0.28, 1.18], *F*[1,210] *=* 10.10, *p* = .002, *η2* = 0.05). Thus, the higher the guilt participants anticipated feeling in a situation in which they have a sick child and go to work the higher the odds that they would avoid such a situation.

Moreover, our previous regression analyses on gender and guilt (see above) show that mothers report anticipating more guilt than fathers. Therefore, we further explore whether mothers were more likely to choose for prioritizing their family and avoid prioritizing their work than fathers. Indeed, a chi-square test confirms that more mothers choose to prioritize family (58.8%) in such a situation than fathers (40.9%; *X*^2^ [1] = 9.95, *p* =.002.

A_m_ = 0.07

Mother’s guilt

Mother’s IAT

*r* = 0.21*

*r* = -0.08

P_mf_ = -0.03

P_fm_ = 0.12

A_f_ = -0.31**

Father’s guilt

Father’s IAT

**Figure 1.** The actor-partner interdependence model, predicting mothers and fathers work-family guilt with their implicit gender stereotypes measured with the gender-career implicit association test (IAT). Standardized regression weights are presented.

A_m_ = effect mother’s IAT on own guilt, A_f_ = effect father’s IAT on fathers’ guilt, P_fm_ = effect of mothers’ IAT on fathers’ guilt, P_mf_ = effect of fathers’ IAT on mothers’ guilt, E_m_ = unexplained portion for mothers’ guilt, E_f_ = unexplained portion for fathers’ guilt. Number of dyads = 68.

**p* < .05; ***p* < .01
