# Supplementary figures and images for: When work–family guilt becomes a women's issue: Internalized gender stereotypes predict high guilt in working mothers but low guilt in working fathers
Source: Br J Soc Psychol. 2022 Sep 13;62(1):12–29. doi: 10.1111/bjso.12575 (PMC10087844; doi:10.1111/bjso.12575)

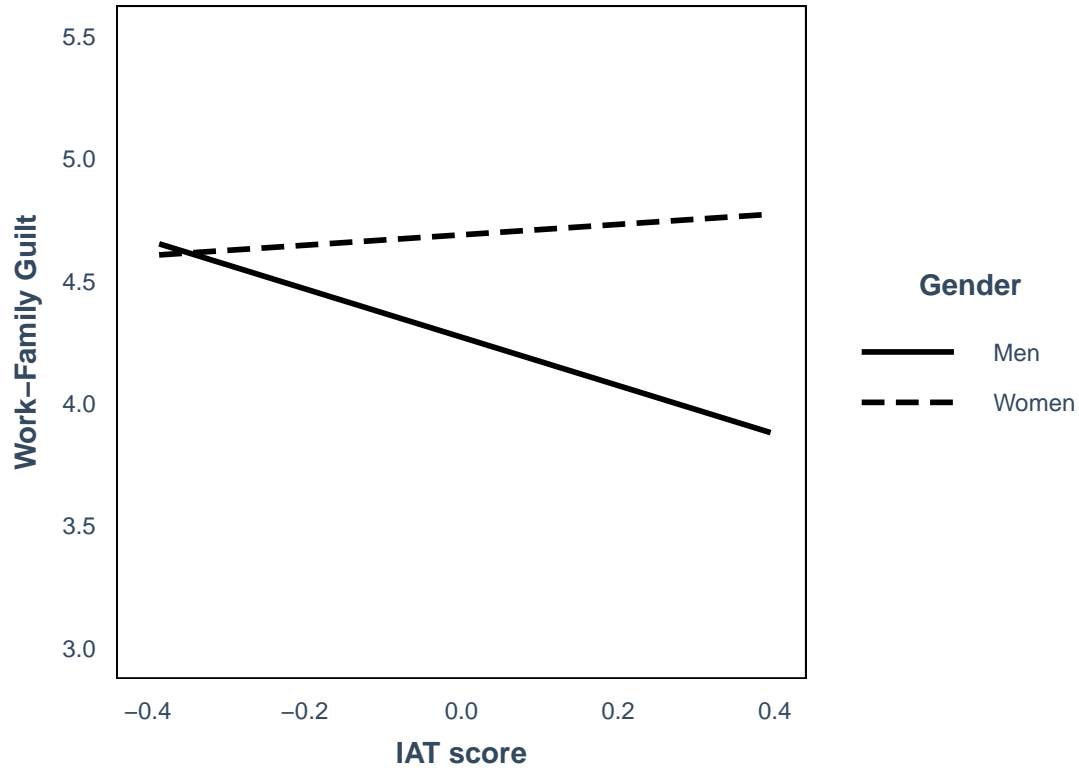

Supplement: Supplementary file 1 — Appendix S1 [file BJSO-62-12-s001.zip › BJSO_12575_Study 1 Figure 1.pdf]

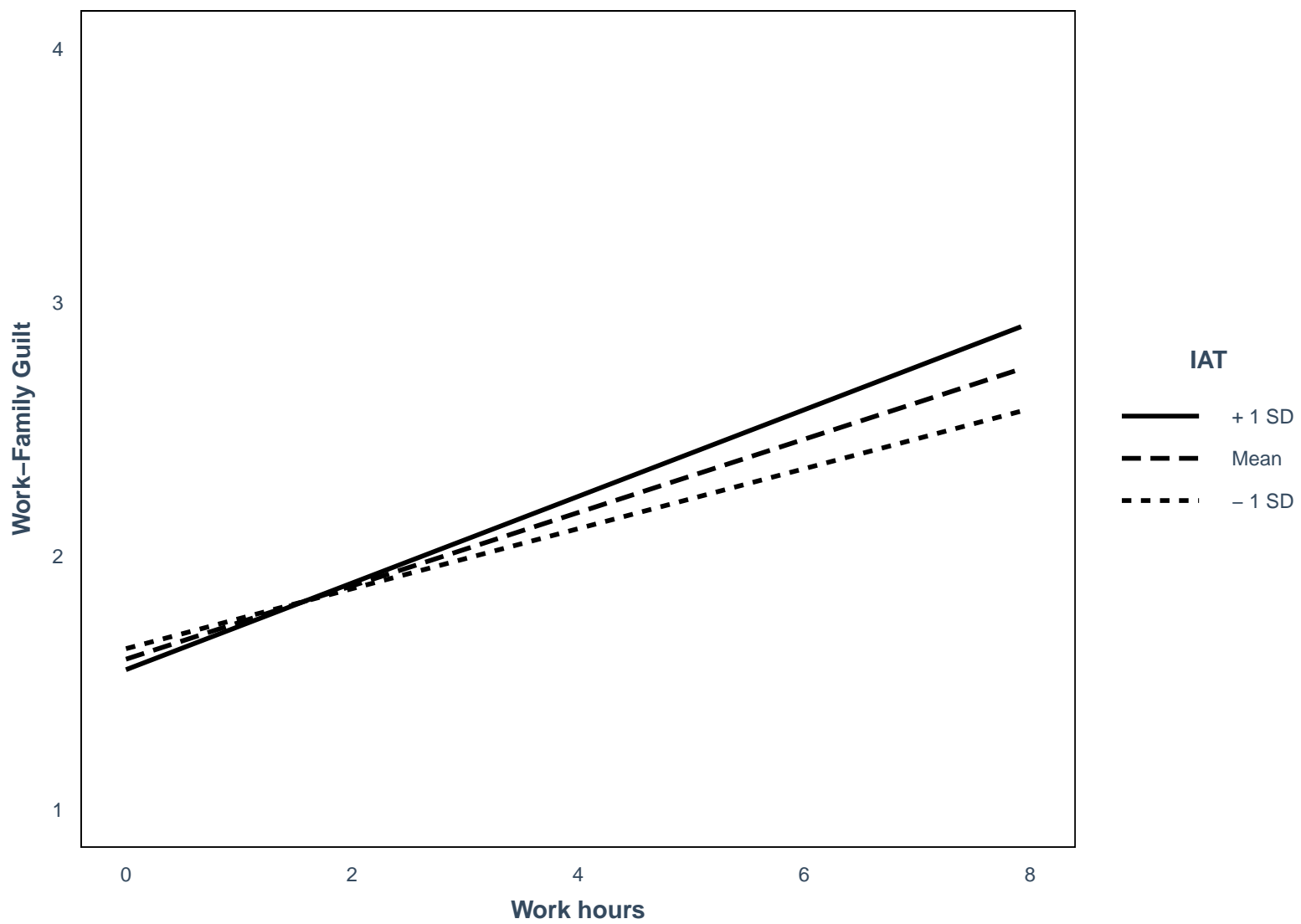

Supplement: Supplementary file 1 — Appendix S1 [file BJSO-62-12-s001.zip › BJSO_12575_Study 2 Figure 2.pdf]
